# Analyzing the delays of target lane vehicles caused by vehicle lane-changing operation

**DOI:** 10.1038/s41598-021-00262-1

**Published:** 2021-11-11

**Authors:** Quantao Yang, Feng Lu, Jun Ma, Xuejun Niu, Jingsheng Wang

**Affiliations:** Department of Traffic Management School, People’s Public Security University of China, Beijing, 100038 China

**Keywords:** Engineering, Mathematics and computing

## Abstract

Vehicle lane-changing on urban roads is the most common traffic behavior, in which the driver changes the direction or increases the speed of the vehicle by changing its trajectory. However, in high-density traffic flow, when a vehicle changes lanes, a series of vehicles following the target vehicle in the target lane will be delayed. In this study, DJI Phantom 4 drones were used to vertically record the traffic on a road section. Tracker software was then used to extract vehicle information from the video taken by the drones, including the vehicle operating speeds, etc. SPSS 22 and Origin analysis software were then employed to analyze the correlations between different vehicle operating parameters. It was found that the operating speed of the first vehicle following the target vehicle in the target lane is related to the speeds and positions of both the target vehicle and the vehicle preceding it. Under the condition of high-density traffic flow, when the target vehicle is inserted into the target lane, the speed of the vehicles following the target vehicle in the target lane will change. To model this process, the corresponding Sine and DoseResp models were constructed. By calculating the delays of vehicles following the target vehicle in the target lane, it was concluded that the overall delay of the fleet is 3.9–9.5 s.

## Introduction

On urban roads, lane-changing (LC) is one of the most common driving tasks. Drivers change lanes to adjust their driving direction and speed. When the traffic density is low, the lane change of a vehicle is helpful for increasing the driver's expected driving speed; however, the lane change of a vehicle under the condition of high-density traffic flow will affect the driving speed of the vehicles following the target vehicle in the target lane (FOL)^1,2^. It has been pointed out in previous research that when the traffic flow in the target lane is 1000–1500 pcu/h and the acceleration of the LC vehicle is 0.1–2.0 m/s^2^, the impact on the speed of the other vehicles in the target lane is the greatest^2^.

To better analyze the impact of LC under high-density traffic flow (0.9 > V/C > 0.8) on urban roads, this work primarily analyzes the speed and delay of the vehicles following the target vehicle. Moreover, the Sine and DoseResp models are used to analyze the variation of the vehicle speed based on traffic wave theory. To simplify the calculation process, the integral is used to calculate the vehicle delay.

The LC of the target vehicle will affect the speed of the vehicles following it. In this study, the relative distance between vehicles was considered as a key influencing factor of the model when investigating the speed changes of vehicles following the target vehicle, and the distance between vehicles of 5.5 m was considered as a key point. It was found that when the distance between vehicles is less than 5.5 m, the vehicle following the target vehicle tends to drive at a constant speed or decelerate; when the distance between vehicles is greater than 5.5 m, the vehicle following the target vehicle tends to first accelerate. To prevent the target vehicle from entering, the speed of the following vehicle gradually decreases when the target vehicle is forcibly inserted.

In urban road traffic, the number of vehicle lane changes far exceeds the number of traffic accidents. Nevertheless, in the study of the consequences of vehicle lane changes, researchers have mainly investigated the severity of traffic accidents caused by vehicles changing lanes, and it has been found that the amount of traffic delay caused by LC far exceeds the number of traffic accidents caused by LC. The delay caused by traffic accidents resulting from vehicle LC is related to the dissipation time of the accident point. The delay of the parking or deceleration of the following vehicles caused by LC is related to the LC time of the target vehicle, which is also the main content of the present research.

While there have been numerous studies on the LC of vehicles, less attention has been paid to the delay of the vehicles following the target vehicle in the target lane (herein referred to as the delay of the vehicles following the target vehicle) during the lane change of the target vehicle. In the present study, research was conducted from the perspective of vehicle delay. The remainder of this paper is organized as follows. “Literature review” section is the literature review. “Methods” section presents the data collection and data processing methods. “Results” section reports the model construction of the first vehicle following the target vehicle (FOL1) and the results. “Discussion” section provides the discussion, and, finally, “Conclusions” section presents the conclusion of this research.

## Literature review

Under the condition of high-density traffic flow (0.90 > V/C > 0.80) on urban roads, due to the small vehicle clearance, the LC of a vehicle will affect the vehicles following it. Vehicle LC can cause traffic delays^3^, thereby causing vehicles to continually stop and start^4^, deteriorating traffic safety^5^, and causing traffic accidents^6,7^ or traffic congestion^8^. Jula et al.^3^ found that vehicle delays caused by vehicle LC/merging collisions account for one-tenth of all vehicle collision delays. Zheng et al.^6^ determined that LC is an important cause of rear-end and side collisions of vehicles on the road. Additionally, it has been concluded that the number of traffic accidents caused by vehicle LC accounts for 4–10% of total traffic accidents^7^. In 2017, there were 1171 collisions caused by vehicle LC in Australia^9^. Moreover, based on Next Generation Simulation (NGSIM) data, vehicle LC has been identified as the main cause of road congestion and collisions^8^. In 2015, Hou et al.^10^ pointed out that collisions caused by vehicles changing lanes accounted for 5% of total collision traffic accidents, and the resulting fatalities accounted for 7% of total collision fatalities. These researchers analyzed the consequences of LC. When a traffic accident occurs on an urban road section, delays will be induced in the vehicles upstream of the accident.

In previous research on vehicle LC models, in which the traffic safety conditions were considered the most important goal, the minimum safety distance of the vehicle was analyzed by considering the minimum safe distance of vehicle-following and the insertion angle of the vehicle as the constraint conditions^11,12^. Xu et al.^12^ regarded the minimum safe distance between vehicles as the safe LC goal of the vehicle. Su et al.^13^ regarded the anti-skidding and anti-rollover limit states in the stressed state of the vehicle as the approaching safety states of vehicle LC, and proposed a formula for the calculation of the minimum LC distance of a vehicle. Li et al.^14^ proposed a model for the calculation of the minimum safe LC distance based on the safe potential field theory; the model can dynamically represent the minimum safe distance under different speed and acceleration conditions. Moreover, the relative speed of vehicles and the relative distance between vehicles have been considered^15^. For example, Cui et al.^16^ utilized probability theory and traffic flow theory to study the combined characteristics of the vehicle LC probability, and used the headway distance as a judgment condition for the occurrence of an event. Additionally, related research has been conducted in terms of the driver’s characteristics and expected speed^17,18^. Yang et al.^17^ constructed the Microscopic Traffic Simulator (MITSIM) model, which can provide the speed change of a vehicle when changing lanes. Zhang et al.^18^ constructed a multi-regime model of vehicle LC behavior. Wang et al.^19^ discussed the characteristics of the driver during the LC process; by collecting driver behavior and vehicle operation data, they found that the driver’s LC frequency was the highest on urban roads (0.82 times/km). Qiao et al.^20^ used the Fourier transform method to explore the relationships between the driver’s LC behavior and the vehicle speed, the traffic volume, and the driver’s psychophysiology, and found that when the rate of increase of the driver’s heart rate is less than 27%, driving safety is higher. Yang et al.^21^ used the attention balance factor to improve the LC model and accurately grasp the impact of the LC of a vehicle on the vehicles following it; however, while actual LC is a process, the study was based on a certain time point for analysis. Zhu et al.^22^ utilized the steering wheel angle and the rate of change of the steering wheel angle to analyze the duration of emergency LC. Pang et al.^23^ found that the duration of the LC of the target vehicle is 1.5–13.9 s, and the average lane change time was 6.88 s.

## Methods

### Data collection method

**Figure 1:**
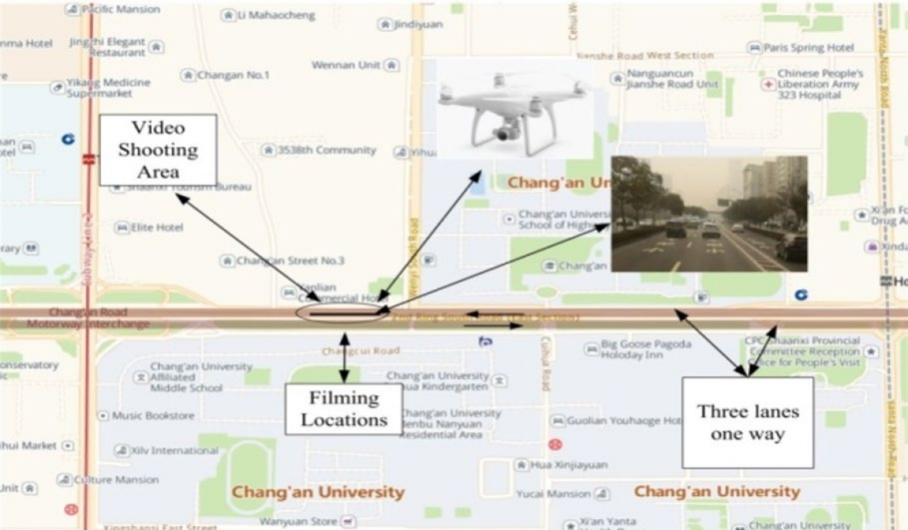
The location of the video collection point. This map was sourced from Google Earth (http://www.gugeditu.net/).

**Figure 2:**
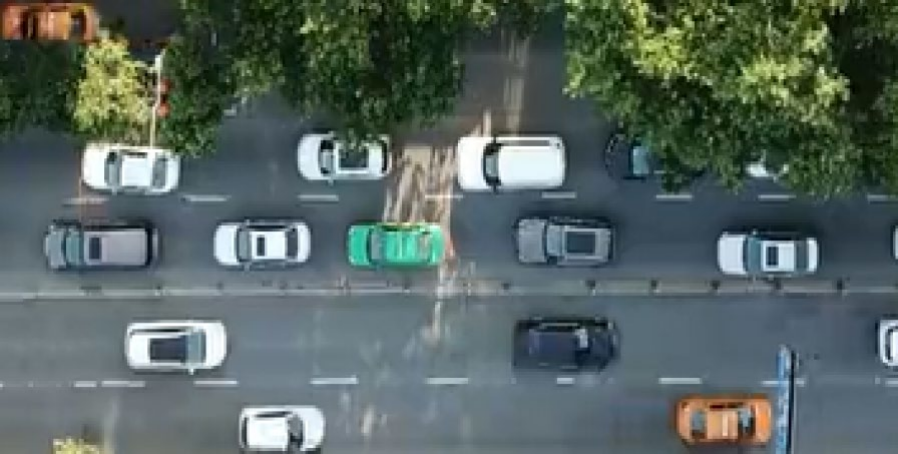
The aerial view of video shooting.

The main road of South Second Ring Road in Xi'an, China was selected as the location for traffic video data collection. The video recording point was the main bridge of Chang'an University. The width of a single lane of this section is 3.75 m. The main road and the auxiliary road of the expressway are separated by guardrails or green belts. Figure 1 presents the location of the video collection point, and the map was sourced from Google Earth (http://www.gugeditu.net/). Figure 2 displays an aerial view of vehicles on the selected road section. DJI Phantom 4 drones were used to vertically record the real-time traffic flow over a length of 200 m. The shooting time was the evening peak hours (17:00–19:00) of a normal working day (August 12, 2019). The DJI Phantom 4 drones used in this research are drone models. Because each video taken by the DJI drones was 15–20 min long, four sets of drones were used for continuous video shooting. After one drone shot a video for 15 min, another drone was flown to the same location. During the shooting process, the drones shot the same scene for a certain period of time, and some of the repeated footage was deleted to ensure a seamless video. It was ensured that each group of drones shot continuously for at least 1 h. Tracker software was used to mark and extract vehicles in the traffic flow and the road information captured by the DJI Phantom 4 drones, including the lane width, vehicle position coordinates, and other information. Table 1 reports a portion of the vehicle data extracted by Tracker software.

**Table 1:** A portion of the vehicle data extracted by Tracker software.

Time (s)	XCTV	YCTV	STV(m/s)	IATV (°)	XCFOL1	SFOL1
57.491	− 16.32	4.47	2.66	15.33	− 0.90	4.63
57.524	− 16.41	4.48	2.69	15.26	− 1.03	4.37
57.558	− 16.50	4.48	2.87	15.18	− 1.19	5.18
57.591	− 16.60	4.47	3.06	15.08	− 1.37	4.65
57.624	− 16.71	4.47	2.86	14.99	− 1.50	4.41
…	…	…	…	…	…	…

These data are located in the authors’ private database. Moreover, the acronyms are defined as follows: XCTV, *x*-coordinate of the target vehicle; YCTV, *y*-coordinate of the target vehicle; STV, speed of the target vehicle; IATV, insertion angle of the target vehicle; XCFOL1, *x*-coordinate of FOL1; SFOL1, speed of FOL1.

### Data processing method

During data analysis, some data are often missing, or some changes in the data are skipped. These problems are inevitable in actual analysis, and abnormal data must be repaired. Common data correction methods include five-point cubic smoothing and locally weighted scatterplot smoothing (LOWESS).

**Figure 3:**
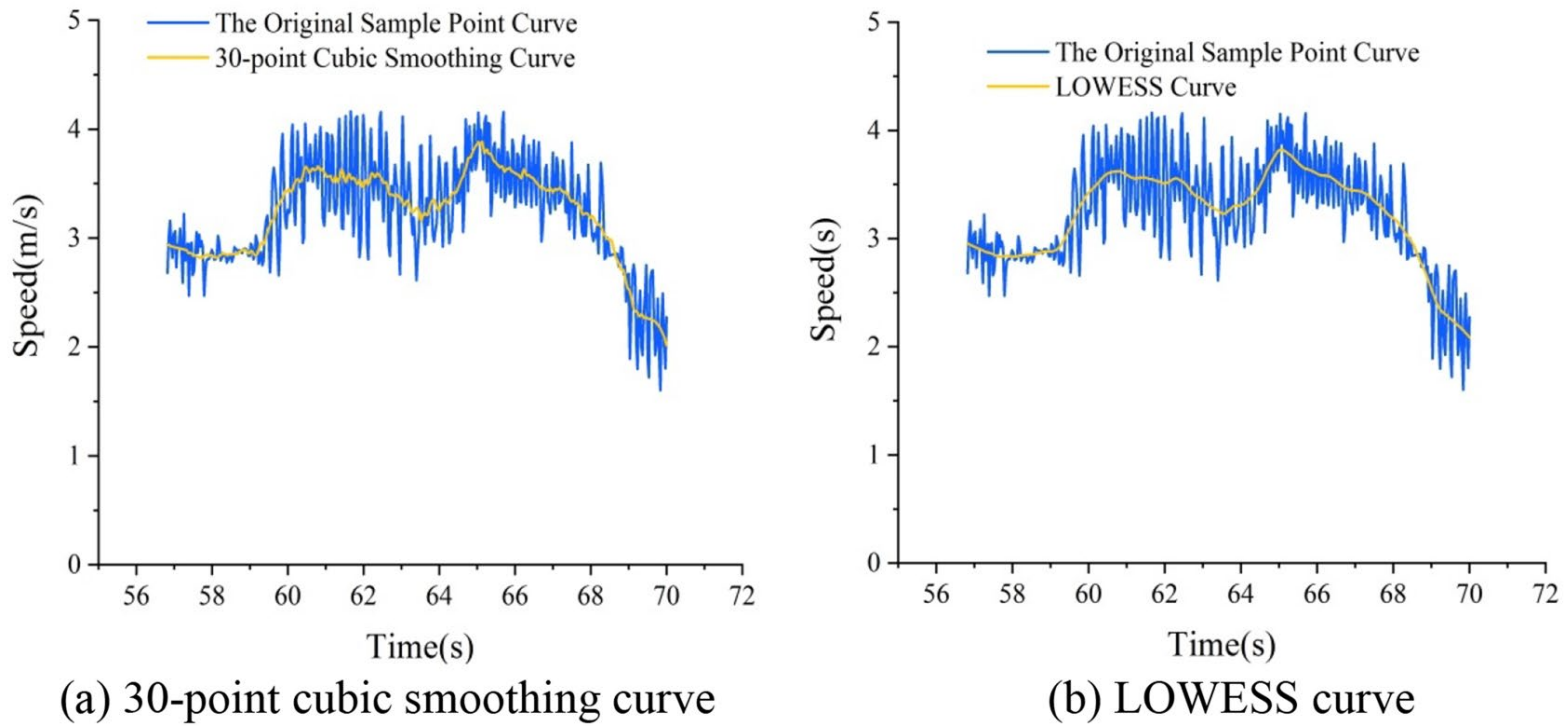
Comparisons between the original data curve and the smooth curves.

Five-point cubic smoothing can sufficiently remove high-frequency random noise in the original data, and is widely used in data processing. Because Tracker software extracts sample points 30 times per second and smooths the data in seconds, the curve of sample points becomes smoother after the five-point fitting is increased to 30-point fitting. Figure 3a presents a comparison between the 30-point cubic smoothing curve and the original data curve.

LOWESS is a non-parametric statistical method for smoothing based on scattered data. It is widely used in data smoothing due to its stability and lower errors. The smoothing concept is mainly to select the center data, take data with a length of *l* on either side of the center data, and conduct a regression to obtain the center value of the regression $\left( {o,\hat{x}} \right)$, where $\hat{x}$ is the fitting curve corresponding to the smooth curve. For all data points *p*, there will be a weighted regression line corresponding to one of them, and the center line of each regression line will be connected to complete LOWESS. Figure 3b presents a comparison between the LOWESS curve and the original data curve.

After the data were smoothed, the accuracy of the smoothed data was verified via the commonly used method of a residual error-relative error test. The residual error is the difference between the observed and predicted values, and can reflect the reliability and periodicity of the data, as given by Eq. (1). Moreover, the relative error can reflect the credibility of the forecasted data, as given by Eq. (2).

1 $$\varepsilon_{i} = x_{i} - \widehat{{x_{i} }},$$

2 $$\Delta \varepsilon_{i} = \frac{{\left| {\varepsilon_{i} } \right|}}{{x_{i} }}$$

where the residual $\varepsilon_{i}$ is the difference between the extracted value $x_{i}$ of the sample at $t_{i}$ and the smooth predicted value $\widehat{{x_{i} }}$, and Δε_i_ is the relative error of the predicted sample at $t_{i}$.

**Figure 4:**
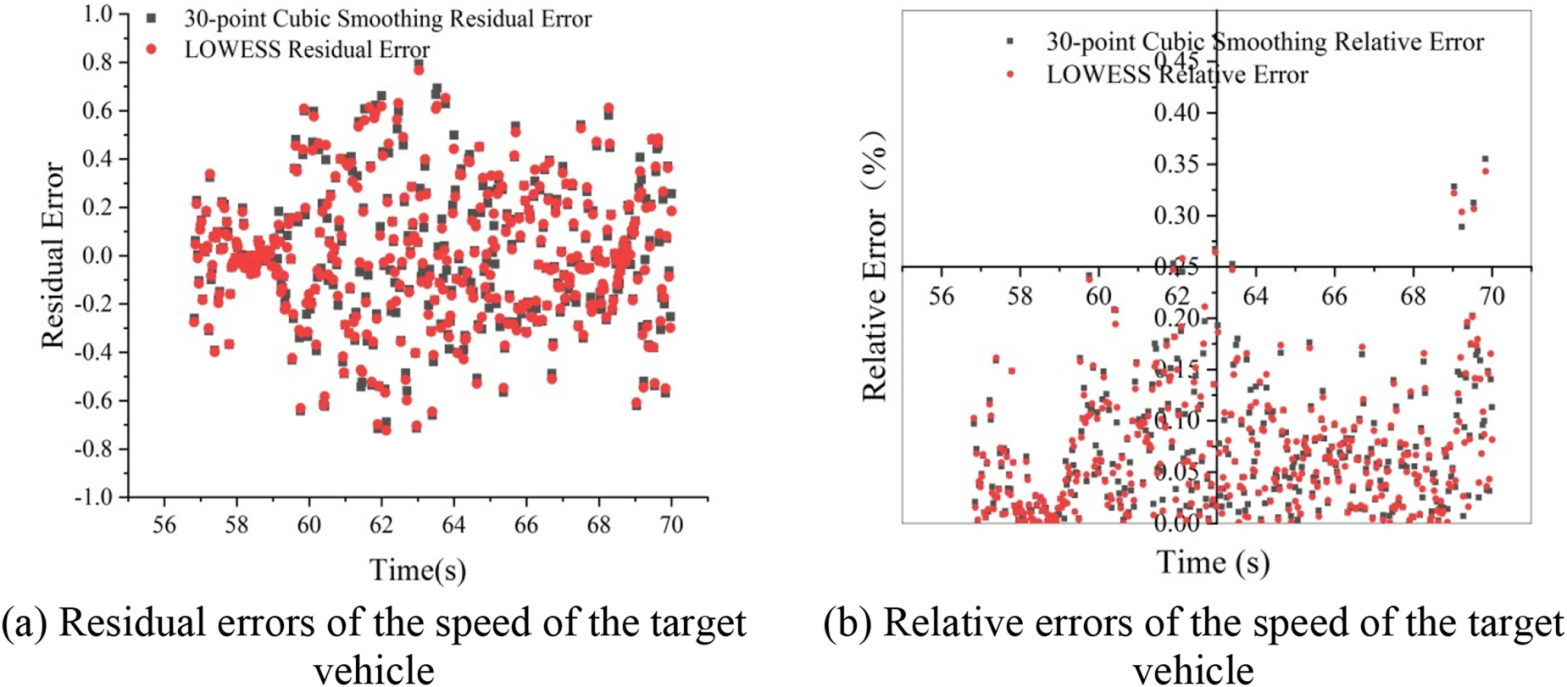
The residual and relative errors of the speed of the target vehicle.

As revealed by Fig. 4, after smoothing the speed data of the target vehicle, it was found that the LOWESS data were slightly better than the 30-point cubic smoothing data in terms of the calculation of the residual and relative errors. To ensure that the data were not distorted, the LOWESS method was used to smooth the survey data.

## Results

### Factors affecting delays

**Figure 5:**
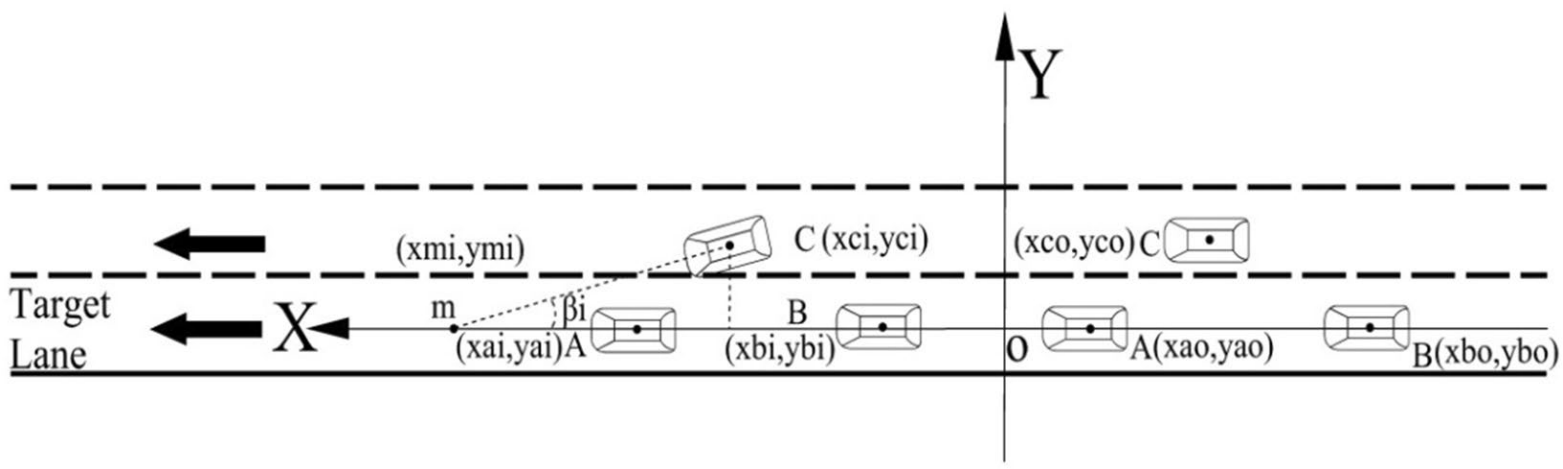
The schematic diagram of the target vehicle changing lanes.

Liu^24^ and Xie^25^ determined the relationship between the distance from the target lane and the speed of the target vehicle during the LC process. Moreover Li et al.^26^ concluded that vehicle LC is also related to the steering wheel angle. When the target vehicle is inserted into the target lane, it is affected by factors such as the distances from and speeds of the vehicles preceding and following it, which causes the target vehicle to adopt different insertion angles and speeds. To better analyze the relationships between the target vehicle and the vehicles preceding and following it, the centerline of the target lane was considered the *x*-coordinate and the direction perpendicular to the centerline was considered the *y*-coordinate to establish a Cartesian coordinate system of the vehicle driving plane, as shown in Fig. 5. At the initial time $t_{o}$, the coordinates of the target vehicle C, the vehicle A preceding target vehicle, and the vehicle B following the target vehicle are respectively $C\left( {x_{co} ,y_{co} } \right)$, $A\left( {x_{ao} ,y_{ao} } \right)$, and $B\left( {x_{bo} ,y_{bo} } \right)$, and the coordinates of vehicles C, A, and B at time *i* are respectively $\left( {x_{ci} ,y_{ci} } \right)$, $\left( {x_{ai} ,y_{ai} } \right)$, and ${ }\left( {x_{bi} ,y_{bi} } \right)$. Additionally, the initial speeds of the three vehicles are respectively $V_{co}$, $V_{ao}$, and $V_{bo}$. The lengths and widths of vehicles C, A, and B are respectively $d_{lc}$ and $d_{wc}$*,* $d_{la}$ and $d_{wa}$, and $d_{lb}$ and $d_{wb}$ (m). When the target vehicle C is inserted into the target lane at a fixed insertion angle, its extended trajectory line intersects the horizontal line of vehicle B at point *m*, and the coordinates of point *m* are ${ }\left( {x_{mi} ,y_{mi} } \right)$. As given by Eq. (3), for the target vehicle to successfully insert into the target lane, the following conditions must be met.

3 $$\left\{ {\begin{array}{*{20}l} {x_{mi} < x_{ai} + \frac{{d_{la} }}{2}} \hfill \\ {x_{mi} > max\left[ {x_{ci} + \frac{{d_{lc} }}{2},x_{bi} + \frac{{d_{lb} }}{2}} \right]} \hfill \\ \end{array} } \right.$$

SPSS 22 analysis software was employed to better analyze the correlations between the influencing factors when the target vehicle is inserted into the target lane. It can be seen from Table 2 that the insertion angle of the target vehicle has significant correlations with its own speed and coordinates, as well as with the speed and coordinates of the vehicles preceding and following it. Moreover, the speed of the vehicle following the target vehicle is related to the coordinates of both the preceding vehicle and the target vehicle, as well as its own coordinates.

**Table 2:** The correlation of various influencing factors.

	XCPRE1	SPRE1	XCTV	YCTV	STV	IATV	XCFOLLL1	SFOL
XCPRE1	1	.545**	.969**	.716**	.087*	.658**	.981**	.331**
SPRE1	.545**	1	.636**	.513**	.553**	.110**	.659**	.586**
XCTV	.969**	.636**	1	.641**	.183**	.573**	.987**	.287**
YCTV	.716**	.513**	.641**	1	.097**	.701**	.685**	.512**
STV	.087*	.553**	.183**	.097**	1	− .171**	.193**	.472**
IATV	.658**	.110**	.573**	.701**	− .171**	1	.577**	.134**
XCFOL1	.981**	.659**	.987**	.685**	.193**	.577**	1	.381**
SFo1	.331**	.586**	.287**	.512**	.472**	.134**	.381**	1

PRE1, the vehicle preceding the target vehicle; XCPRE1, the *x*-coordinate of PRE1; SPRE1, the speed of PRE1.

**Statistically significantly correlated at the .01 level; *Statistically significantly correlated at the .05 level.

### Prediction results of FOL1 speed

**Figure 6:**
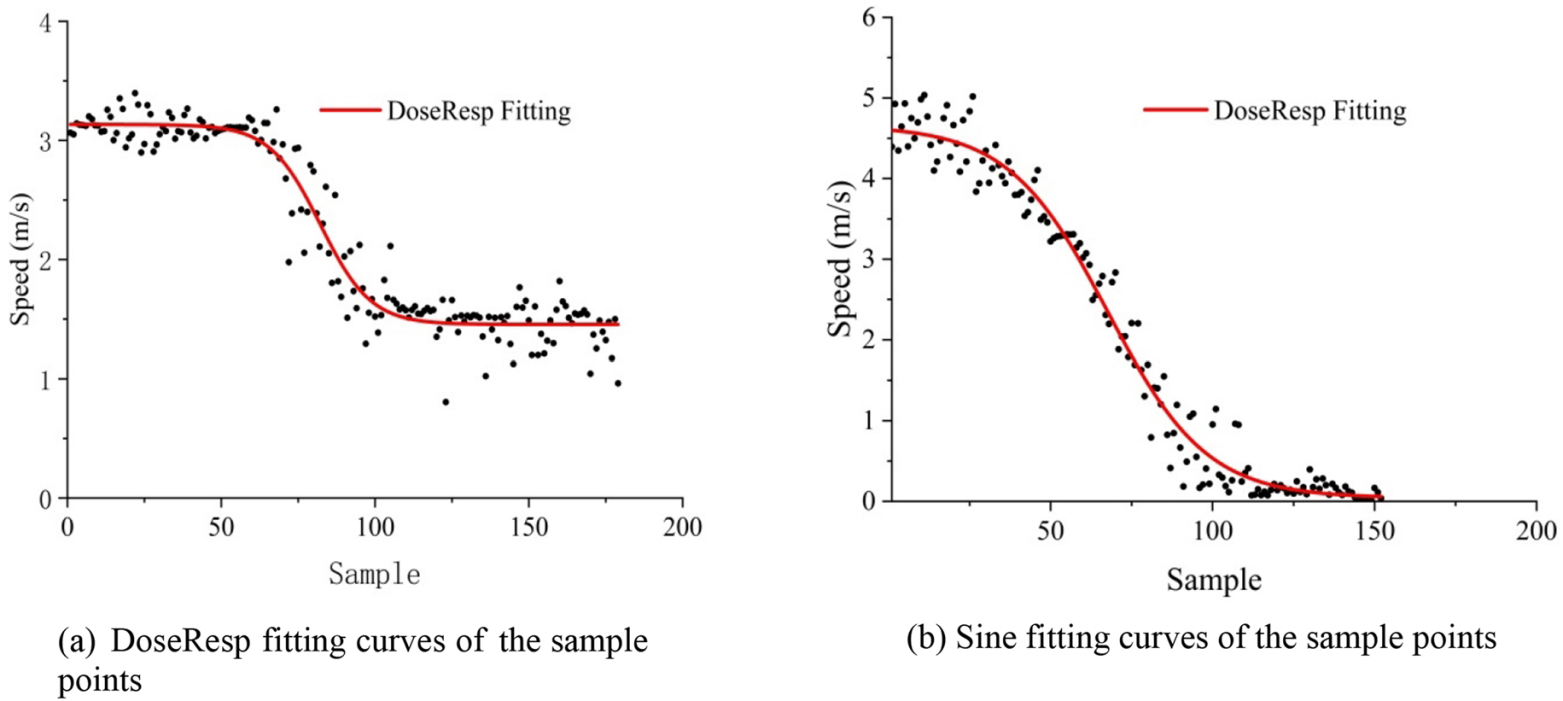
The fitting curves of the sample points.

To better analyze the speed change of FOL1, after fitting 21,440 data points of 10 sets of samples, it was found that the speed changes of six sets of samples conformed to the DoseResp model when the speed was gradually reduced. As shown in Fig. 6a, by fitting these six sets of data, it was found that the speed of FOL1 is related to its own speed at $t_{0}$, its distance from the vehicle preceding the target vehicle, and the total lane change duration $t_{c}$ of the target vehicle. During LC, it is also related to the average insertion angle $\overline{\beta }$ and the initial *y*-coordinate value of the target vehicle. Equation (4) describes the speed curve of FOL1.

4 $$V_{{bi}} = min\left( {\frac{{x_{{pre,i}} - x_{{fol1,i}} }}{{d_{{lb}} }}} \right) + \frac{{v_{{10}} - min\left( {\frac{{x_{{pre,i}} - x_{{fol1,i}} }}{{d_{{lb}} }}} \right)}}{{1 + 10^{{(y \times \left| {x_{{pre,0}} - x_{{00}} } \right| - t_{i}) * \left( { - t_{c} \times \bar{\beta }} \right)}} }},$$

where $V_{bi}$ is the speed of FOL1 at time *i* (m/s), $x_{pre,0}$ is the condition of the preceding vehicle at time $t_{0}$ (m), $x_{fol,0}$ is the condition of FOL1 at time $t_{0}$ (m), $V_{10}$ is the initial speed of FOL1 at $t_{0}$ (m/s), ${ }x_{pre1,i}$ is the condition of the preceding vehicle at time $t_{i}$, ${ }x_{fol1,i}$ is the condition of FOL1 at time $t_{i}$, where ${ } i \in 30t_{c}$, $t_{c}$ is the lane change duration of the target vehicle, and $\Delta {\text{l}}_{i}$ is the distance between vehicles at time $t_{i}$.

Moreover, the speed of FOL1 during the LC process was found to first increase, then decrease, then increase again. As presented in Fig. 6b, after fitting the other four groups of sample points, it was found that the change in the vehicle speed at this time conforms to the Sine fitting model. Equation (5) describes the speed curve of FOL1.

The speed of FOL1 changes from moment to moment due to the influence of the target vehicle. In the process of the target vehicle gradually entering the target lane, its distance from FOL1 is decreasing, and the speed of FOL1 is gradually decreasing. The Sine model fluctuates under the influences of the vehicle distance and speed. During the process of function fitting, the function offset is related to the distance between the vehicles preceding and following the target vehicle and the length of the following vehicle; the phase is related to the distance between the vehicles, the ordinate, and the time of the LC of the target vehicle; the amplitude is related to the initial speed of the vehicle following the target vehicle.

5 $$V_{bi} = max\left( {\frac{{x_{pre,i} - x_{fol,i} }}{{d_{lb} }}} \right) + v_{10} \times {\text{sin}}\left( {pi \times \frac{{t_{i} - y_{i} \times \Delta l_{i} }}{{30t_{c} }}} \right)$$

**Figure 7:**
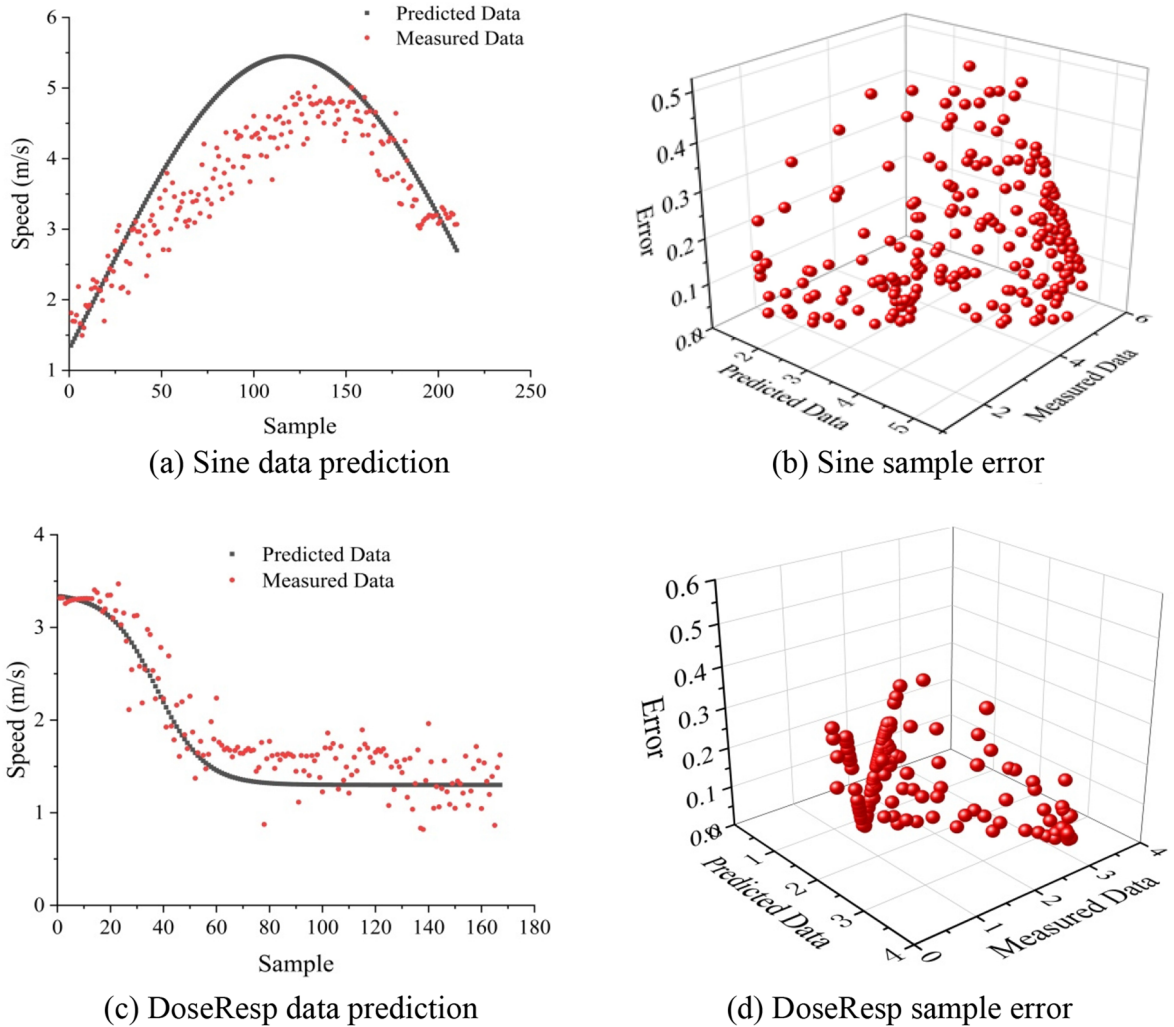
The sample point prediction and error analysis.

To verify the accuracy of the speed change model of FOL1, Eqs. (4) and (5) were used to predict the vehicle speed. The predicted data were then compared with the original data, and the relative error was used to analyze the fitting results of the sample. Figure 7 presents the vehicle speeds predicted by the two methods and the relative errors of the samples.

Two sets of new data were selected. The first set included 235 sample points; at the initial moment, the distance between FOL1 and the preceding vehicle was 6.1 m, and the data were fitted using Eq. (5). The second set included 138 sample points; at the initial moment, the distance between FOL1 and the preceding vehicle was 4.8 m, and the data were fitted using Eq. (4). By comparing the speeds predicted by Eqs. (4) and (5) with the original data at each moment, it was found that the distributions of the two groups of predicted data were consistent with the original data, and the relative error of 90% of the two groups of prediction data was below 0.3, indicating that the numerical prediction was reasonable.

Sine fitting is suitable for the situation in which the distance between the vehicles preceding and following the target vehicle is large. Due to the large distance, during the insertion of the target vehicle, FOL1 will accelerate to shorten the distance between the target vehicle and the preceding vehicle. After insertion, the distance between FOL1 and the target vehicle becomes increasingly closer, and FOL1 decelerates in order not to collide with the target vehicle. Therefore, SFOL1 increases and then decreases. After completing the LC process, SFOL1 tends to stabilize. When the distance between the vehicles preceding and following the target vehicle is small, during the insertion of the target vehicle, FOL1 first decelerates at a constant speed or directly decelerates until the speed of the target vehicle stabilizes after the LC process is completed. When the distance between the vehicles preceding and following the target vehicle is *l* ≥ 5.5 m, the Sine model better fits the speed of FOL1, as FOL1 will increase its speed to prevent the target vehicle from inserting. When the distance between two adjacent vehicles is *l* < 5.5 m, the target vehicle is forced to insert, and FOL1 is forced to decrease its speed. In this case, DoseResp better fits SFOL1.

### Results of FOL delay

When the target vehicle C is inserted into the target lane, the speed of vehicle B is constantly changing, and the distance traveled by vehicle B within the interval $t_{b}$ from its initial speed to when the speed stabilizes is $S_{b}$. When calculating the vehicle driving distance, the randomness of the vehicle speed and the inconsistency of the initial speed complicate the solution process. The calculation must therefore be simplified to ensure accuracy.

6 $$S_{b} = \mathop \smallint \limits_{0}^{{t_{b} }} v_{bi} dt \approx S_{tb} - S_{t0}$$

Because the target vehicle C will cause the speed of vehicle B to decrease when it inserts into the target lane, the delay time of vehicle B over the same driving distance is $t_{by}$ (s). When the target vehicle C is inserted into the lane, the total delay of all vehicles following the target vehicle is $t_{y}$ (s).

7 $$t_{by} = \frac{{v_{b0} \times t_{b} - S_{b} }}{{v_{b0} }}{ }$$

8 $$t_{y} = \mathop \sum \limits_{i = 1}^{n} t_{iy}$$

In Eq. (8), $t_{iy}$ is the delay time of the *i*-th vehicle following the target vehicle, (s), and *n* is the number of delayed vehicles following the target vehicle. By observing 10 sets of field data, the target vehicle was found to have a relatively large impact on the first to fifth vehicles immediately following it, and to have relatively little or no impact on the vehicles after the fifth vehicle.

**Figure 8:**
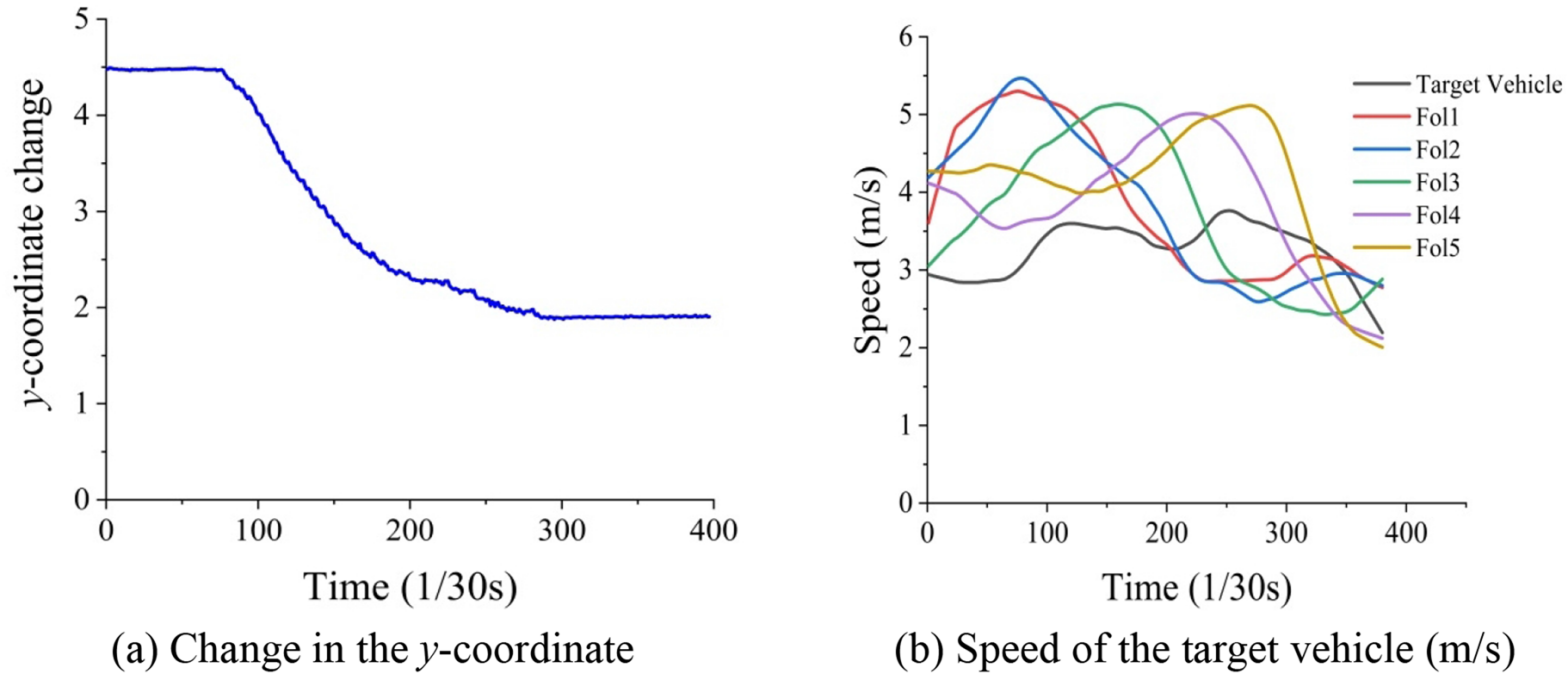
The vehicle information. In (**a**), the horizontal axis represents the LC time of the target vehicle, and the vertical axis represents the change of the coordinate of the target vehicle. Subfigure (**b**) presents the speed change of the vehicles in a set of survey samples.

Taking the target vehicle and the vehicles following it as a set of samples, the delays of FOL during the LC process were calculated. It can be seen from Fig. 8a that the target vehicle started to change lanes from the 72nd point, and this process ended at the 280th point. It took about 6.93 s for the target vehicle to change lanes from the beginning to the end of the LC process.

From Fig. 8b, it can be seen that the speed of FOL1 decreased from the 140th point to the 233rd point, and the speed then began to stabilize. According to Eq. (6), the driving distance of the target vehicle in the target lane can be determined. The driving distance of FOL1 was 10.34 m, i.e., $S_{b} { }$ = 10.34 m. The speed of FOL1 at the 140th point was 5.38 m/s, the speed change duration was 3.1 s, and the theoretical driving distance was 16.68 m. At this time, according to Eq. (7), the delay time of FOL1 was $t_{by }$ = 1.2 s, $t_{dy} { }$ = 1.1 s, $t_{ey }$ = 1.1 s, $t_{fy}$ = 1.1 s, and $t_{gy}$ = 0.8 s.

To calculate the delay of each group of LC vehicles, the number of vehicles affected by each group of LC vehicles was first analyzed. Then, according to Eq. (8), the longest delay was calculated as 9.5 s, and the shortest delay was calculated as 3.9 s. After averaging the 10 sets of delay data, the average delay of each group of vehicles was determined to be 6.6 s.

## Discussion

When the target vehicle is changing lanes, the speed change of FOL follows the vehicle-following theory. The speed change of the second vehicle following the target vehicle (FOL2) will occur slightly later than that of FOL1*.* After the target vehicle changes lanes, the speeds of the target vehicle and the vehicle following it will eventually converge. This delay exhibits a gradually decreasing trend, is subject to the vehicle-following characteristics studied by Xu^27^, and is consistent with delay and transitivity. The speed change of FOL1 is related to the vehicle distance; when the vehicle distance *l* ≥ 5.5 m, the speed change will conform to the Sine model, and when the vehicle distance *l* < 5.5 m, the speed change will conform to the DoseResp model.

Traffic fluctuation theory was used in the vehicle delay analysis in this study. It was more intuitively demonstrated that the LC of the target vehicle will indeed cause a series of delays of the vehicles following it, and the delay will be between 3.9 and 9.5 s. To ensure the overall operating efficiency of the road under the condition of high-density traffic flow (0.9 > V/C > 0.85 in this study), the unnecessary LC of vehicles should be prohibited. Zhang et al.^28^ found that the LC time is 5.38 s, which is also in line with the research results of Han et al.^29^ who found that the vehicle LC time is in the range of 1.7–10.3 s.

## Conclusions

This research investigated the impact of vehicle lane-changing (LC) on the speed of vehicles following the target vehicle in the target lane under the condition of high-density traffic flow on urban expressways (0.9 > V/C > 0.8). According to the curve of the speed change of the vehicles following the target vehicle, when the current distance between the first vehicles preceding and following the target vehicle is *l* ≥ 5.5 m, the speed change of the first vehicle following the target vehicle in the target lane (FOL1) conforms to the Sine model, and when the current distance between the vehicles is *l* < 5.5 m, the speed change is consistent with the DoseResp model. It was also concluded that the lane change of the target vehicle will cause a delay of 3.9–9.5 s for the vehicles following the target vehicle. Based on the results of this study it was concluded that in the state of high-density traffic flow, vehicle LC will affect the running speed and delay of the vehicle FOL1.thus, under this condition, vehicle LC should be prohibited to ensure higher traffic efficiency. This research provides a theoretical reference for the analysis of LC of driverless vehicles. To successfully complete a lane change, a driverless vehicle must comprehensively consider the running state of the vehicle following it, not only to improve its own running speed, but also to reduce the impact on the vehicle behind it. In future research, the reasonable speed and insertion angle of vehicle LC in the optimal travel time will be determined based on the overall operating efficiency of adjacent vehicles. The research results obtained in this work were based on actual data combined with software analysis. However, although the characteristics of the driver of the first vehicle following the target vehicle will affect the behavior of the target vehicle, they were not considered in the present study. Moreover, the driving characteristics of the driver of the target vehicle will reflect the actual vehicle operating speed. These factors will be considered in future research.

## Data Availability

The datasets generated and analyzed during the current study are available from the corresponding author on reasonable request.
